# A preclinical animal study to evaluate the operability and safety of domestic one-way endobronchial valves

**DOI:** 10.3389/fmed.2024.1293940

**Published:** 2024-05-01

**Authors:** Yang Jiao, Sen Tian, Jian Liu, Xiaping Shen, Qin Wang, Xiang Li, Wei Zhang, Yuchao Dong, Yonghua Li, Chong Bai, Haidong Huang

**Affiliations:** ^1^Department of Respiratory and Critical Care Medicine, The First Affiliated Hospital of Naval Medical University, Shanghai, China; ^2^Department of Respiratory and Critical Care Medicine, No. 906 Hospital of the Chinese People’s Liberation Army Joint Logistic Support Force, Ningbo, China; ^3^Department of Radiology, The First Affiliated Hospital of Naval Medical University, Shanghai, China; ^4^Department of Respiratory and Critical Care Medicine, General Hospital of Central Theater Command of Chinese People's Liberation Army, Wuhan, China

**Keywords:** one-way endobronchial valve, bronchoscopy, emphysema, lung volume reduction, safety, animal, operability

## Abstract

**Purpose:**

To evaluate the operability and safety of bronchoscopic domestic one-way endobronchial valves (EBV) on animals.

**Methods:**

Nine pigs were randomly assigned (2:1) to receive domestic one-way EBV (the experimental group, *n* = 6) and Zephyr^®^ EBV (the control group, *n* = 3). Routine blood tests, arterial blood gases, and CT scans of the lungs were performed 1 day pre-procedure in addition to 1 week and 1 month post-procedure to assess changes in blood markers and lung volumes. At 1 month post-procedure, the animals were sacrificed, followed by removal of all valves via bronchoscopy. Pathological examinations of critical organs were subsequently performed.

**Results:**

A total of 15 valves were placed in the experimental group and 6 valves were placed in the control group, without serious complications. Routine blood tests and arterial blood gas examinations at 1 day pre-procedure, 1 week post-procedure, and 1 month post-procedure did not differ significantly in both groups. No EBV displacement was noted under bronchoscopy, and the valve was smoothly removable by bronchoscope at 1 month post-procedure. At 1 week post-procedure, varying degrees of target lung lobe volume reduction were observed on lung CT in both groups. Lung volume reduction was achieved at 1 month post-procedure in both groups, without significant statistical difference. Although 3 cases in the experimental group and 1 case in the control group developed varying degrees of pneumonia, the inflammatory response did not increase over time during the experimental period. Pathological examination revealed no significant abnormal changes in the critical organs for both groups.

**Conclusion:**

Our results demonstrate that domestic EBV is safe and reliable for endobronchial application in general-grade laboratory white pigs. The safety of domestic EBV is similar to that of Zephyr^®^ EBV, with good ease of use and operability. This kind of domestic EBV can meet the safety evaluation requirements for animal testing.

## 1 Introduction

Chronic obstructive pulmonary disease (COPD) is a common condition with an estimated overall global prevalence of 10.3%, which is now one of the top three causes of death worldwide (1, 2). Emphysema is a form of COPD that is defined by abnormal and permanent enlargement of the airspaces distal to the terminal bronchioles and is associated with the destruction of the alveolar walls. The destruction of alveolar walls causes loss of elastic recoil, airway closure during exhalation, and air trapping in distal air spaces. Severe emphysema substantially affects patients’ quality of life and is highly susceptible to a series of complications such as respiratory failure, which can be life-threatening (3). Most traditional interventions, such as oxygen inhalation combined with antispasmodic drugs and rehabilitation exercises, have minimal effects and fail to adequately control disease progression in patients with advanced emphysema (4–7). Lung volume reduction surgery (LVRS) was first proposed in the 1950s (8), and subsequent clinical studies confirmed that LVRS improves exercise tolerance and quality of life in patients with severe emphysema (9); however, its widespread clinical use has been limited by the recent considerable mortality rate associated with surgery and potential serious postoperative complications (10, 11). In the National Emphysema Treatment Trial, the most common complications of LVRS include reintubation, arrhythmias, and mechanical ventilation for more than 2 days (12, 13); less common complications include intraoperative myocardial infarction, deep venous thrombosis, pulmonary embolism, and wound infection (14).

Bronchoscopic lung volume reduction (BLVR) refers to a technique developed to treat hyperinflation due to emphysema via a flexible bronchoscope, which causes atrophy and fibrosis of the target lung, resulting in regional lung volume reduction and achieving the same therapeutic goal as that of lung volume reduction surgery (15, 16). One-way endobronchial valves (EBV) have been designed for bronchoscopic placement based on the hypothesis that they will allow air and mucus to exit the treated area but do not allow air to re-enter (17). This treatment can facilitate atelectasis of the emphysematous, hyperinflated lung distal to the EBV (18). The US Food and Drug Administration (FDA) has approved EBV for bronchoscopic placement in lung regions with little to no collateral ventilation to treat patients with hyperinflation associated with severe emphysema (19). The main EBV listed in China is the Zephyr^®^ EBV (Pulmonx Inc., Redwood City, CA, United States), however it is expensive. The development of domestic EBV products has major clinical significance in reducing the economic burden on patients and improving prognosis. In this study, safety and operability were compared between EBV products manufactured by Beijing Saishute Medical Devices Co., Ltd. and those manufactured by Pulmonx Corporation using an *in vivo* animal model to provide a reference for future clinical research.

## 2 Materials and methods

### 2.1 Materials

#### 2.1.1 Experimental animals

In total, nine laboratory white pigs of general grade were selected as the experimental animals and were purchased from Wujiang Tianyu Biological Technology Co., Ltd. (license number: SCXK(Su) 2016-0006). The experimental and control groups were enrolled at a 2:1 ratio. The experimental and control groups were comprised of 6 and 3 animals, respectively.

#### 2.1.2 Product specifications

**Figure 1:**
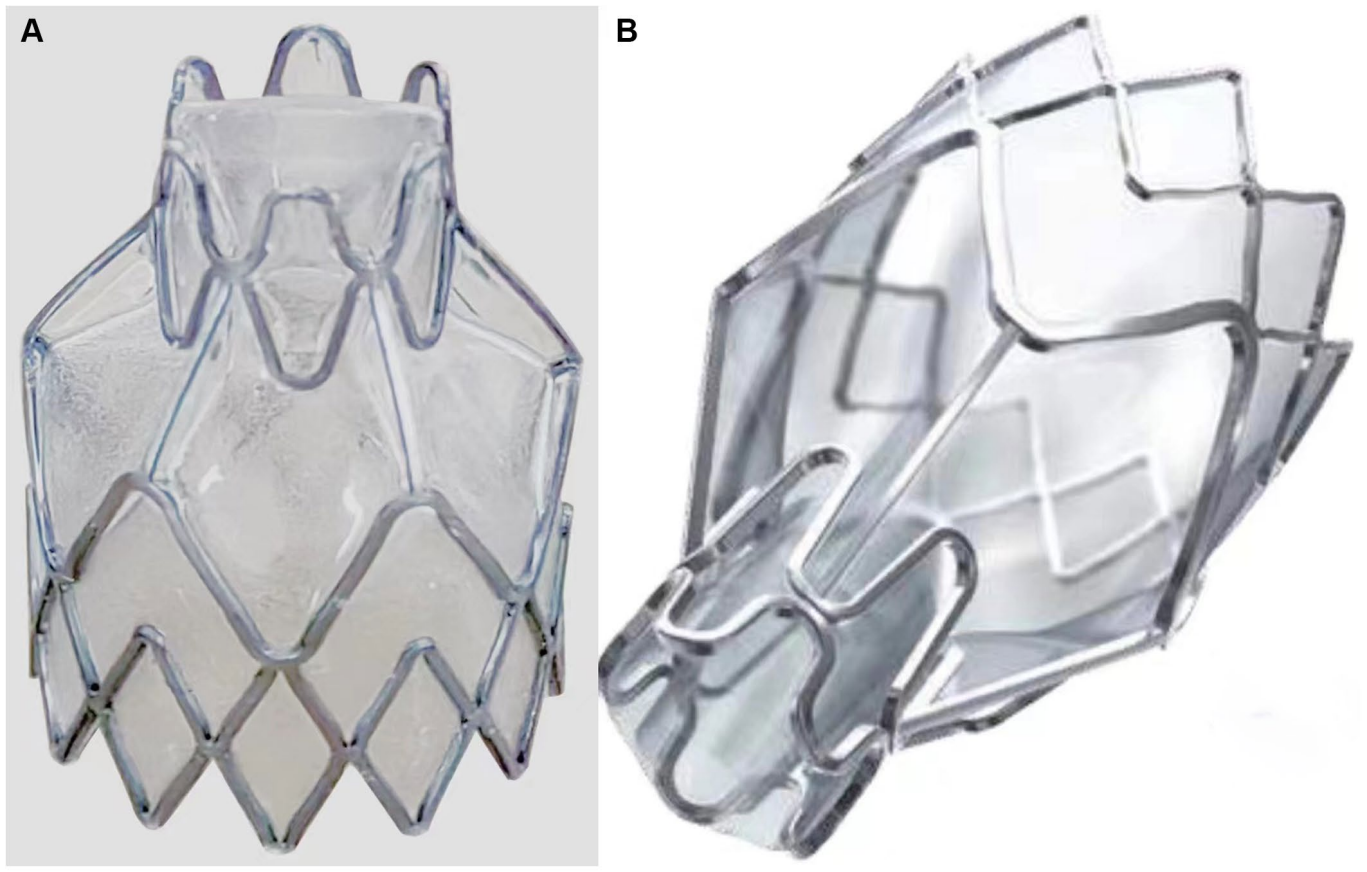
Comparison of two groups of endobronchial valves (EBV) styles. **(A)** The EBV of experimental group with 6-segment waveform structure at the near end. **(B)** The EBV of control group with 5-segment waveform structure at the near end.

The product consists of a frame made of nickel-titanium alloy and a one-way duckbill valve made of silicone rubber. A silicone rubber film is used to cover the frame surface. As shown in Figure 1, the EBV of experimental group with 6-segment waveform structure and control group with 5-segment waveform structure have a different shape due to the manufacturing processes. However, the overall dimensions (such as the total length, support length, protection length, distal diameter, and proximal diameter) and the radial force of these two EBV are similar. The product is sterilized with ethylene oxide and has an expiration date of 2 years. The product contains four specifications: EBV-4.0 (experimental group), EBV-5.5 (experimental group), EBV-TS-4.0 (control group), and EBV-TS-5.5 (control group), each of which is appropriate in different diameter of bronchus.

#### 2.1.3 Other equipment and materials

Other equipment and materials included a multi-parameter vital sign monitor (PHILIPS MP70, United States), automatic blood cell analyzer (Mindray BC-5000), cardiac defibrillation monitor (PHILIPS Heartstart MRx, United States), anesthesia machine (Nanjing Pu’ao Medical Jinling-013536A), blood gas analyzer (GEM 4000, United States), Computed tomography (CT) machine (Canon Tax-303A, Japan), electronic bronchoscope (Zhuhai Vision), foreign body forceps (Alton Medical Instruments, Shanghai), extendable suction tube (Dräger ErgoSt*r, Germany), lobar ventilation function tester (Pulmonx, United States), catheter and connector specific for lobar ventilation function tester (Pulmonx, United States), and EBV delivery catheter (Pulmonx, United States).

### 2.2 Methods

#### 2.2.1 Randomization scheme

This study adopted a randomized, blinded design. The outer packaging of EBV manufactured by Beijing Saishute Medical Devices Co., Ltd. and Pulmonx Corporation International Medical Devices Co., Ltd. was removed or covered and placed in blank envelopes. The envelopes containing the device products were numbered after sequence reordering. During the recording and evaluation process, the devices in both the experimental and control groups were represented by the device serial numbers, and the operators and evaluators were completely blinded to this information. According to the randomization method, the serial numbers were randomly assigned using computerized randomization software. A randomly generated table of device serial numbers was provided to the operator who completed the surgery. This information was unblinded to the statistical analysis.

#### 2.2.2 Surgical procedure

Preoperative handling of the experimental animals, including preoperative anesthesia and preparation of the preoperative surgical area, were conducted in accordance with the Standard Operating Procedure of MINCAL Medical Research (MK-OR-205 Capture and Anesthesia of Laboratory Pigs). In the experimental animals, the EBV was placed in the target lung lobe using aseptic techniques. During surgery, animals were maintained under general anesthesia, and their hemodynamics, electrocardiogram, body temperature, and blood oxygen saturation were monitored in real time. The surgical procedure was performed as follows: first, anesthesia was induced by intramuscular injection of a combination of tiletamine (3 mg/kg), zolazepam (3 mg/kg), and ketamine (5 mg/kg), followed by maintenance of anesthesia by inhalation of 60% oxygen and 1 to 3% isoflurane. Tracheal intubation was then performed to establish the respiratory passage and marginal ear vein. A peripheral vein was catheterized for delivery of fluids and drugs as needed. A femoral artery puncture was performed to monitor the ambulatory blood pressure. Electrocardiograms, preoperative vital signs, and body position were recorded. The target lung lobe for endoscopic occlusion was determined based on preoperative lung CT. An electronic flexible bronchoscope was inserted via tracheal intubation, and the lobar ventilation function tester and special detection catheter were connected to reach the target position through the bronchoscope. A balloon detection catheter was used to connect the tester to detect ventilation of the target lobe bypass. Subsequently, an EBV delivery device with a valve at the end was used to deliver the valve to the opening of the target bronchus for valve release. The opening and closing conditions of the valve were good, and no leakage occurred around the valve during the procedure. The bronchoscope was withdrawn after the valve was firmly placed and probed with biopsy forceps. Antibiotics were administered to prevent infection after surgery.

#### 2.2.3 EBV performance evaluation

Quantitative scoring was performed on a scale of 1–10, with one being the worst, ten being the best, and five being intermediate. Three aspects were evaluated: placement position (according to the instructions for use, the device must be placed distally to the target airway ridge and obstruct the target airway), mobility (the device can be removed after being correctly placed in a section of the trachea), and migration (after the device has been placed in the trachea for a period of time, the distal end should be in the original ridge position and be able to obstruct the corresponding airway). A corresponding evaluation was performed by the surgical operators of this experiment based on the actual situation during use.

#### 2.2.4 Follow-up indicators

Three follow-up time points were set: 1 day pre-surgery, 7 days post-surgery, and 1 month post-surgery. All experimental animals were subjected to a final follow-up and sampling. CT of the lung was performed at 7 days and 1 month post-surgery to assess the EBV status and changes in lung volume. Blood samples were collected 7 days and 1 month post-surgery for routine blood tests and blood gas examinations. Bronchoscopy was performed 1 month post-surgery to observe the working state of the valve and the occurrence of any displacement.

Lung lobe segmentation and volume analysis were performed by using Synapse 3D (FUJIFULM, Version 4.4 EU). Firstly, DICOM data was imported. Secondly, the left and right lungs were separated, and lung lobe segmentation was accomplished through the “Lung analysis/Airway” module automatically. If automatic lung separation or lobe segmentation failed or was inaccurate, computer-aided manual lung separation or lobe segmentation was performed. Both lungs were separated according to manually drawn separated lines, or lobe segmentation was accomplished following manually outlining lobe margins on multiple CT slices (The overlay ratio was 0.32, each lung lobe was outlined every 12 frames from head to tail and the middle part was automatically filled. If there was a problem in the filled part, we manually modified). Thirdly, Synapse 3D calculated lung and lobe volume automatically.

At the end of the experimentation, the experimental animals were sacrificed by intravenous injection of 10 mL pentobarbital sodium (390 mg/mL) and phenytoin (50 mg/mL) (Euthasol) for overall necropsy, including examination of the external surface of the animal carcass, orifices, mucous membranes, and cranial, thoracic, and abdominal cavities. The heart, liver, spleen, lungs, kidneys, brain tissue, and organs were isolated and excised. The examination results were recorded. The lungs were removed intact and inflated slightly. Then, the lungs were visually observed and palpated to determine the site of valve placement and to observe the area of atrophy. Lung tissue was obtained from the atrophic area, non-atrophic area, and contralateral normal lung tissue. After fixation and staining, pathological changes at the placement site and normal lung tissue adjacent to the thoracic, bronchial, blood vessel walls, and alveolar tissue were observed under a microscope 1 month after valve placement in experimental animals.

### 2.3 Statistical analysis

Statistical analyses were performed using SPSS24 software. Normally distributed data with equal variance are expressed as the mean ± standard deviation (*x* ± *s*). Analysis of variance (ANOVA) was used to compare the means between multiple groups. For skewed data or uneven variance, the data are expressed as median (interquartile range), and the rank-sum test was used to compare the means of multiple groups. *p* < 0.05 was considered statistically significant.

## 3 Results

### 3.1 General information

A total of 9 cases of experimental white pigs were planned for this animal study, and 9 cases were actually operated on. The weight range of animals was 35–40 kg. The first placement was performed on October 17, 2021, and the last animal reached its endpoint on November 19, 2021. All animals were successfully placed with EBV according to the experimental protocol. The surgery proceeded smoothly with no abnormal deaths or adverse events during the postoperative rearing period. The usage distribution of each EBV in the target lung lobes in each experimental animal is presented in Table 1.

**Table 1:** Details of placed EBV.

Sequence	Serial number	Placement site	Number of placed EBV	Batch of EBV	Specification of EBV
1	P-1534	RUL	1	21,090,001	5.5
2	P-1523	Lingula of LUL	1	504,602-V6.2	5.5
3	P-1535	RML	1	504,602-V6.2	5.5
4	P-1537	Upper division of LUL	1	21,090,001	5.5
5	P-1536	LLL	2	21,090,001	4.0
6	P-1538	LLL	4	504,389-V6.2 504,602-V6.2	4.0/5.5
7	P-1541	RUL	2	21,090,001	4.0/5.5
8	P-1540	Basal segment of RLL Inner Anterior Posterior Outer	7	21,090,001	4.0 4.0 4.0 4.0/5.5
9	P-1542	RML	2	21,090,001	4.0

EBV, Endobronchial valves; RUL, Right upper lobe; LUL, Left upper lobe; RML, Right middle lobe; LLL, Left lower lobe; RLL, Right lower lobe; RML, Right middle lobe.

### 3.2 Electronic bronchoscopic imaging data

**Figure 2:**
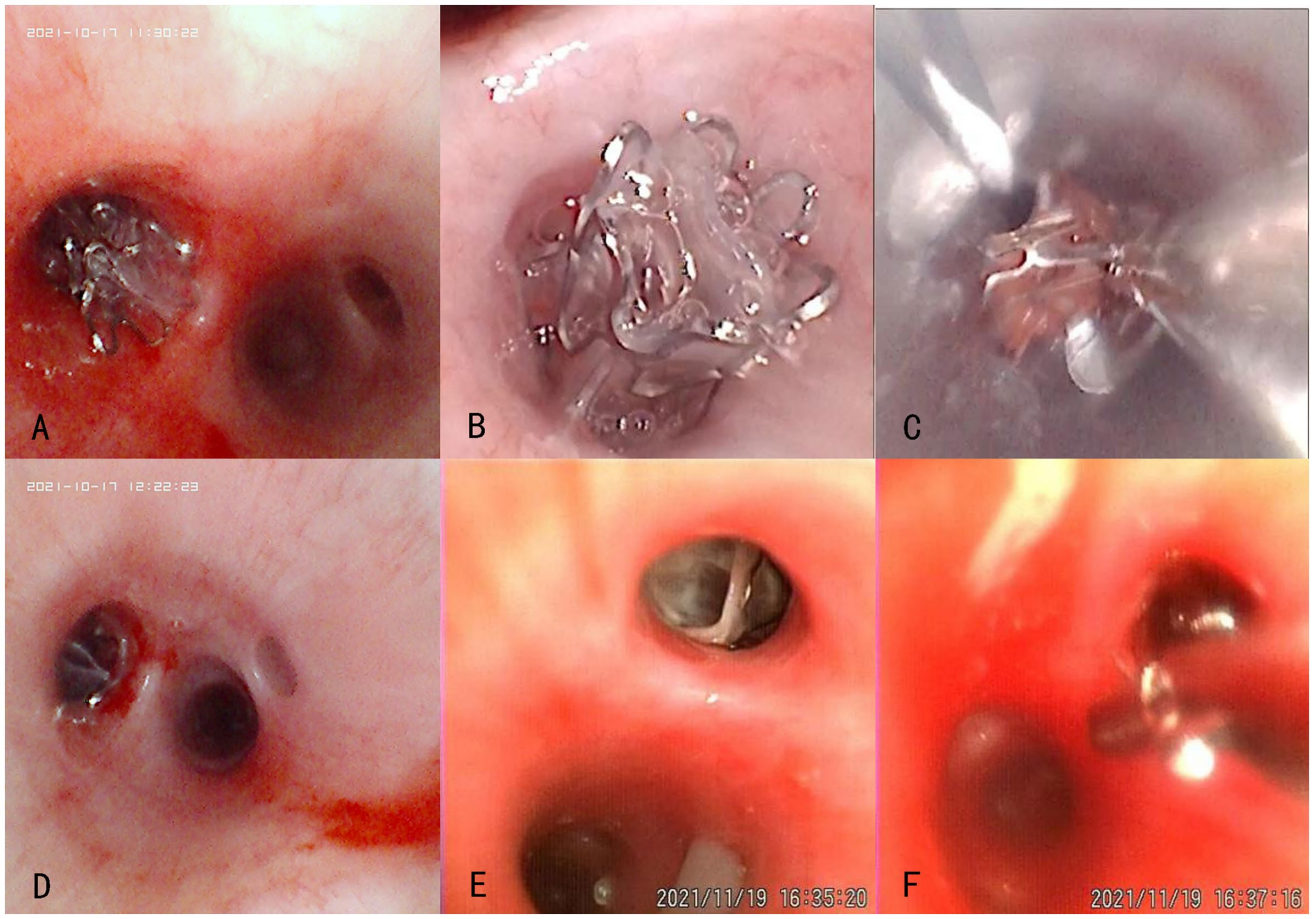
Bronchoscopic views of the experimental (No. P-1536, **A–C**) and control (No. P-1538, **D–F**) groups at different time points after the product placement. **(A,D)** Placement in the left lower lobe during surgery; **(B,E)** Condition around the valve opening 1 month post-surgery; **(C,F)** Smooth removal at 1 month post-surgery.

Bronchoscopic images revealed that the valve was well positioned and could completely obstruct the target airway in the experimental group. Furthermore, the valve was not displaced and was removable with foreign body forceps 1 month post-surgery. Two animals in the experimental group exhibited a small amount of localized granulation at the distal end after valve removal, and a small number of secretions did not affect the ventilation of the lumen. In the control group, the valve was well placed during surgery and completely obstructed the target airway. One control animal had a Zephyr® 4.0 valve that fell off 7 days post-surgery. The remaining valves were not displaced and were removable with foreign body forceps 1 month post-surgery. One control animal exhibited a small amount of granulation, mucosal edema, and secretions at the distal end of the valve after valve removal. Bronchoscopic images of the experimental and control groups are presented in Figure 2.

### 3.3 Evaluation of lung volume reduction using lung CT images

**Figure 3:**
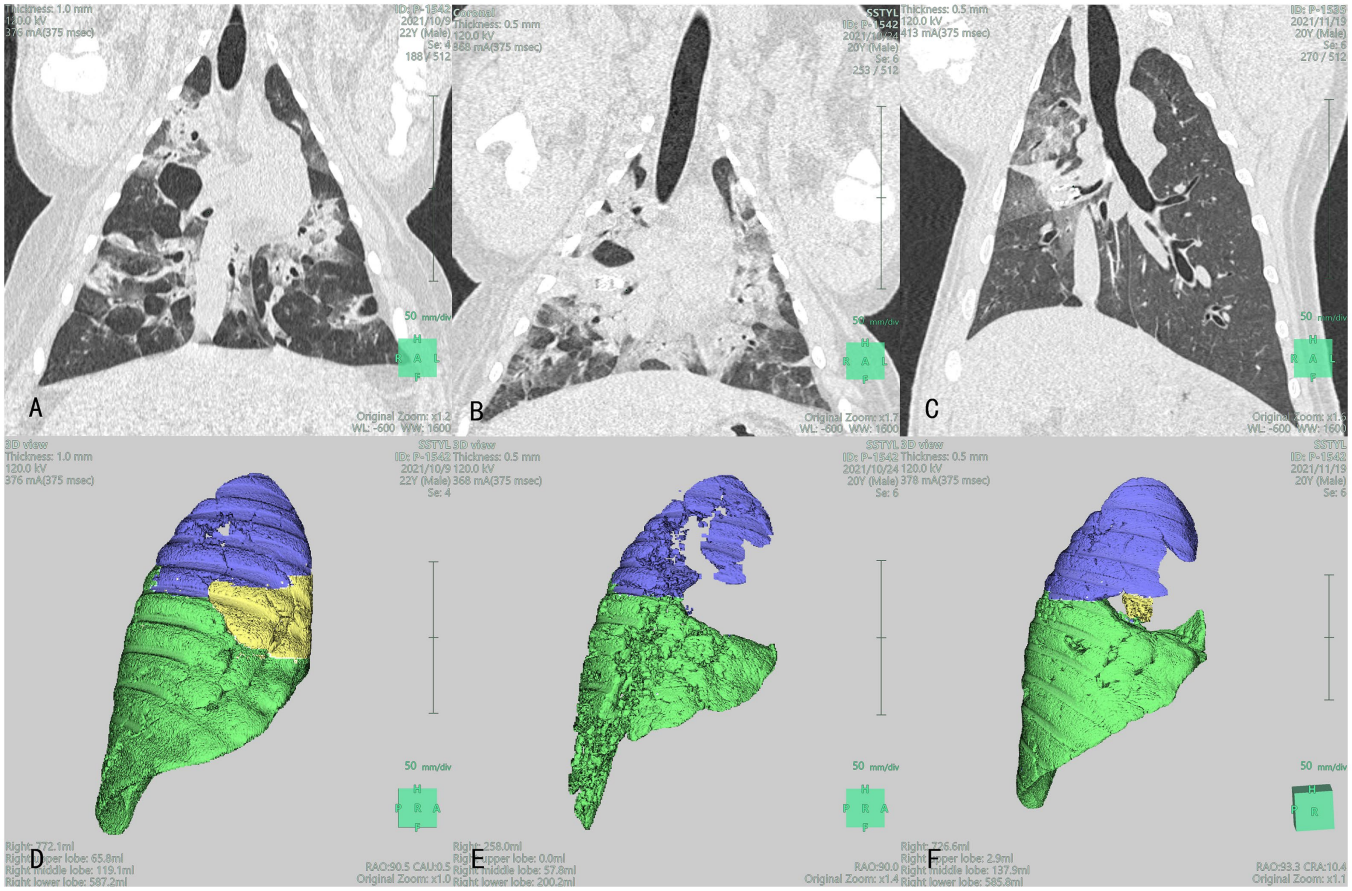
Lung CT **(A–C)** and 3D reconstruction **(D–F)**: Blue, yellow, and green section represent the right upper lobe (RUL), right middle lobe (RML), and right lower lobe (RLL), respectively images of the lungs of the experimental group at different time points after product placement in animal No. P-1542: **(A)** 1 day pre-surgery; **(B)** Visible volume reduction in the RML at 7 days post-surgery; **(C)** Visible volume reduction in the RML at 1 month post-surgery; **(D)** The volume of RML was 65.8 mL 1 day pre-surgery; **(E)** The volume of RML was 0 mL at 7 days post-surgery; **(F)** The volume of RML was 2.9 mL at 1 month post-surgery.

**Figure 4:**
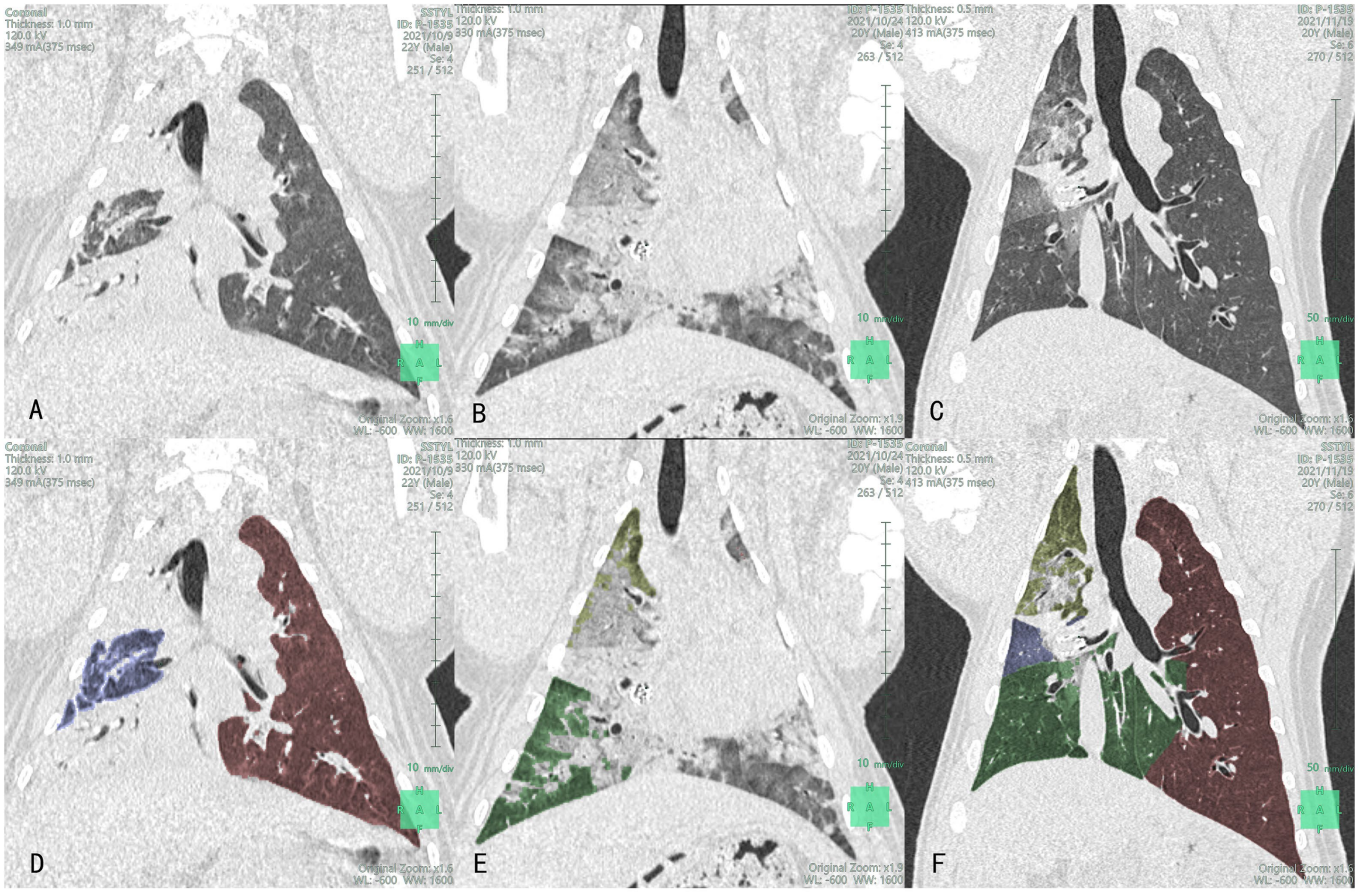
Lung CT **(A–C)** and lobe segmentation **(D–F)**: Yellow-green, blue and green section represent the right upper lobe (RUL), right middle lobe (RML), and right lower lobe (RLL), respectively images of the lungs of the control group at different time points after product placement in animal No. P-1535: **(A)** 1 day pre-surgery; **(B)** Visible volume reduction in the RML at 7 days post-surgery; **(C)** Visible volume reduction in the RML at 1 month post-surgery; **(D)** The volume of RML was 53.8 mL 1 day pre-surgery (RUL and RLL were mostly consolidated); **(E)** The volume of RML was 0 mL at 7 days post-surgery; **(F)** The volume of RML was 12.6 mL at 1 month post-surgery.

CT scans of the lungs revealed that the volume of the lung lobes at the placement site was reduced in the experimental group at 7 days and 1 month post-surgery. In one animal in the experimental group, volume reduction was not evident at 7 days post-surgery but was visible at 1 month post-surgery. In the control group, volume reduction of the lung lobes at the placement site was visible at 7 days and 1 month post-surgery. In one control animal, volume reduction was not evident at 7 days post-surgery but was visible at 1 month post-surgery. Lung volume reduction was achieved in both experimental and control groups, with no significant difference between the groups. CT images of the experimental and control groups are presented in Figures 3, 4, respectively.

Segmentation of the targeted lobe/segment (left propria upper lobe in animal No. P-1537 and dorsal segment of right lower lobe in animal No. P-1536) could not be achieved in the experimental group, and that of the left lingual lobe in animal No. P-1523 could not be achieved in the control group. Moreover, the preoperative right upper lobe of animal No. P-1534 could not be separated due to insufficient CT slices. Therefore, the volume assessment of these four experimental animals was mainly based on qualitative analysis. Nearly complete target lobe/segment atelectasis was found at 7 days post-surgery in animals No. P-1534 and No. P-1536 in the experimental group, and partial lobe recruitment was found at 30 days post-surgery in both animals. Lobe volume was apparently reduced at 7 days and 30 days post-surgery in both animals No. P-1537 in the experimental group and animal No. P-1523 in the control group and nearly no lobe recruitment was found except for animal No. P-1538 in the control group, lobe volume was apparently reduced in four other animals at 7 days post-surgery, and there was no significant increase in lobe volume at 30 days post-surgery (CT, lobe segmentation, and 3D reconstruction images of the experimental and control groups are presented in Figures 3, 4, respectively). Furthermore, lobe volume reduction was achieved in both groups, while there were no significant differences in lung lobe/segment volume at preoperation, 7 days, and 30 days post-surgery between the two groups.

### 3.4 Histopathological evaluation of major organs

**Figure 5:**
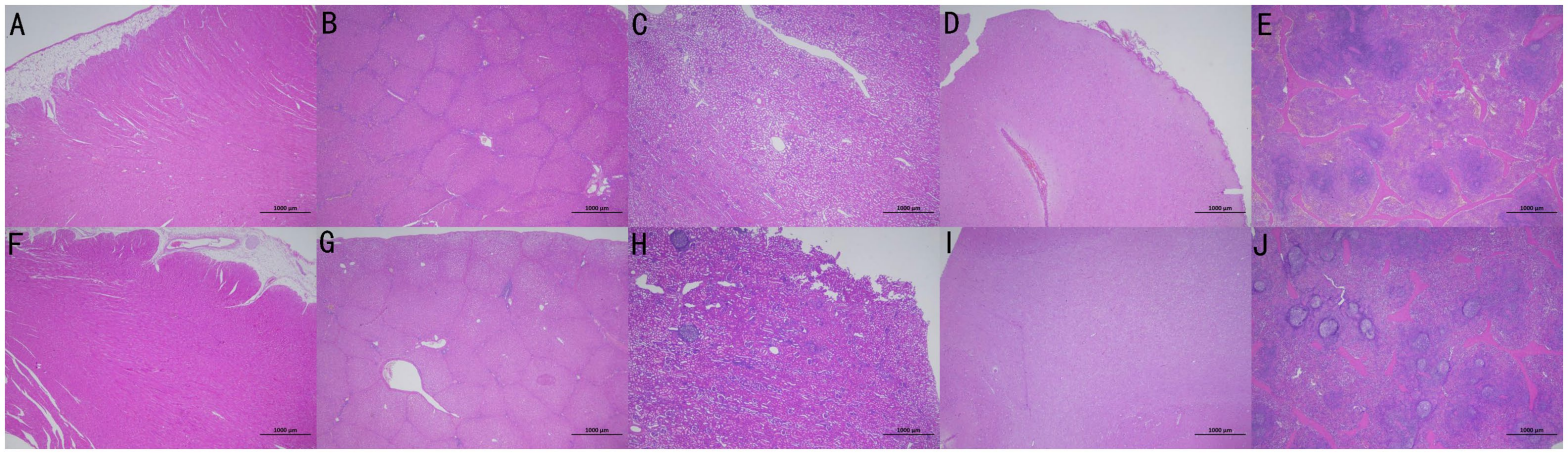
Pathological specimens of organs in the experimental (No. P-1536, **A–E**) and control (No. P-1523, **F–J**) groups were presented in the order of heart, liver, kidney, brain, and spleen from left to right.

Pathological necropsy was performed on the experimental animals after euthanasia. The pathological specimens of the organs in the experimental and control groups are presented in Figure 5. No significant pathological changes were observed in the thoracic cavity of animals in the experimental and control groups. Atrophy of the lung tissue was observed at the distal end of the valve *in vivo*. The abdominal cavity was intact, and no significant pathological changes were noted in the abdominal wall and peritoneum, heart tissue, kidney, spleen, and brain tissue. The lung tissues of the animals in the experimental and control groups exhibited varying degrees of lung infection. Alveolitis was observed in 3 cases in the experimental group and 1 case in the control group. Inflammatory infiltration around the airways was present in 5 cases in the experimental group and 1 case in the control group. All of these animals had lung infections present on imaging before surgery, which were not exacerbated by the placement of EBV during the experimental period. No significant differences in inflammation were observed between the groups.

### 3.5 Laboratory indicators

Routine blood test results of the experimental and control groups were within the normal range 1 day pre-surgery, 7 days post-surgery, and 1 month post-surgery, with no significant differences between the groups (Table 2). No significant difference was observed in oxygen partial pressure between the experimental and control groups at 1 month post-surgery.

**Table 2:** Comparison of main laboratory indicators between the experimental and control groups.

	Follow-up time	Experimental group	Control group	*p*
pH	1 day pre-surgery	7.38 ± 0.11	7.29 ± 0.05	All >0.05
	7 days post-surgery	7.28 ± 0.11	7.25 ± 0.12	
	1 month post-surgery	7.39 ± 0.03	7.46 ± 0.04	
PaO_2_(mmHg)	1 day pre-surgery	384.20 ± 181.86	258.00 ± 142.91	
	7 days post-surgery	61.83 ± 14.62	61.33 ± 8.99	
	1 month post-surgery	45.17 ± 10.16	217.33 ± 234.54	
PaCO_2_(mmHg)	1 day pre-surgery	60.40 ± 16.58	77.00 ± 13.14	
	7 days post-surgery	72.67 ± 14.46	85.00 ± 20.61	
	1 month post-surgery	52.50 ± 2.75	44.67 ± 3.40	
WBC (10^9^/L)	1 day pre-surgery	19.01 ± 3.33	20.19 ± 2.11	
	7 days post-surgery	19.47 ± 1.39	19.71 ± 3.65	
	1 month post-surgery	17.58 ± 2.26	15.02 ± 0.23	
N(10^9^/L)	1 day pre-surgery	5.28 ± 1.80	8.21 ± 1.68	
	7 days post-surgery	5.49 ± 2.69	5.77 ± 3.93	
	1 month post-surgery	6.12 ± 1.90	4.80 ± 1.87	

### 3.6 EBV performance evaluation

The evaluation results of the experimental and control groups were 8–10 and 7–10 points, respectively. No significant differences were observed in the overall evaluation between the experimental and control groups (Table 3).

**Table 3:** Evaluation of EBV manipulation.

	Experimental group *N* = 6	Control group *N* = 3	*p*
Placement site	9.33 ± 0.75	9.00 ± 0.00	All >0.05
Mobility	9.67 ± 0.75	9.67 ± 0.47	
Migration	9.83 ± 0.37	9.00 ± 1.41	

EBV, Endobronchial valves.

## 4 Discussion

EBV, as an implantable device designed to allow secretions and gas to be discharged from lung tissue but prevent gas from entering lung tissue at the distal end of the valve, eventually leads to atelectasis of the treated lobe, provided if the absence of collateral ventilation between the treated lobe with EBV and the untreated ipsilateral lobe (20). Based on positive outcomes of the treatment on symptoms, pulmonary function, exercise capacity and quality of life in multiple randomised controlled trials (2), EBV treatment has gained popularity over the course of the last decade and currently served as the most widely used lung volume reduction method for select patients with severe emphysema (21–24).

EBV products mainly include duckbills and umbrella valves. The initial design of the Pulmonx EBV system was that of a nitinol skeleton and a silicone body with a “duckbill” valve at the proximal end. The most recent version of this EBV, known as the Zephyr^®^ valve, maintains the “duckbill” mechanism. Several randomized trials and a multicenter registry have reported modest improvements in symptoms and lung function after BLVR with the placement of Zephyr^®^ EBV (25–27), but with a risk of pneumothorax of 25 to 30 percent (28–30). The Spiration EBV uses an umbrella-shaped nitinol-framed prosthesis with a synthetic polymer cover (31). The flexible nitinol frame allows for the valve to maintain contact with the airway wall and prevent air from passing inwards while allowing mucus and air to escape. This creates a one-way valve effect with the intent of redirecting airflow to more normal areas and/or inducing atelectasis of the emphysematous area blocked by the valve. However, subsequent studies in this series found that this type of EBV did not significantly improve the patient’s quality of life and the 6-min walking test (32, 33). Therefore, duckbill valves are mainly used in the market not only simple in structure and easy to install and maintain, but also have more use value.

Pigs are an established model system for testing products intended for human use. The trachea and tissue organs of pigs are anatomically and physiologically similar to those of humans; thus, many clinical studies have chosen pigs as reliable models for EBV placement (34). The objectives of this study were only accomplishable using live animals, and no *in vitro* model or non-animal testing was available as an alternative. The pathophysiology of emphysema lesions cannot fully recapitulate the human disease process, as they are not easily modeled. Therefore, animal models were not employed in this regard. In addition, the main purpose of our animal experiments was to assess the safety of the device through *in vivo* testing and to identify potential complications. Accordingly, using normal small pigs was sufficient for our purposes. The entire study complied with the relevant animal testing regulations of the National Medical Products Administration (35).

The nine animals enrolled in the study exhibited varying degrees of pneumonia in their lungs 1 day pre-surgery, 7 days post-surgery, and 1 month post-surgery. The inflammatory response did not increase over time during the experimental period. The experimental animals were in a good condition before, after, and at the end of the experimentation. Indeed, the animals presented with normal dietary habits and stools and did not exhibit clinical manifestations of pneumonia, such as fever or respiratory symptoms. Hence, CT findings were considered old lesions or related to the animals’ rearing environment rather than due to device placement. Laboratory test indicators and pathological sections of the major organs at different follow-up time points throughout the experiment also confirmed no significant differences between the two groups. For performance evaluation of the EBV, the influence of subjective interpretation of scores was avoided due to strict blinding procedures. No significant differences were observed in final scores based on operator experience between domestic and imported EBV, except for animal No. P-1538, whose EBV was coughed up in the control group, lobe/segment volume was apparently reduced in other animals at 7 days post-surgery. However, lobe/segment volume increased to various degrees in some animals at 30 days post-surgery. The valve was well positioned, completely obstructed the target airway, and was instantly removed at 30 days post-surgery. Therefore, the reason may be that pigs are hyperactive, causing temporary dysfunction of the one-way valve during rearing. Moreover, due to different degrees of poor ventilation or consolidation before surgery, there may be certain errors in automatic and computer-assisted artificial lung volume extraction.

Compared with the imported EBV, the domestic EBV has a 6-segment symmetrical waveform network structure, so the radial force is more uniform, and the fit with the bronchial wall is stricter. The radial force of the domestic EBV is smaller than that of the imported EBV, and a smaller radial force can obtain firmer fixation and reduce the risk of granulation growth. Compared with imported EBV, the proximal diameter is 15% larger, accelerating lung volume reduction. In addition, in the first silicone rubber dipping process, the product flap and the frame part of the film under specific conditions improve the production efficiency. Simultaneously, the overall valve coating is more uniform and stronger, reducing the risk of film damage and leakage. Because of these advantages, the domestic EBV is demonstrated good operability and effectiveness in this study.

The current study contains several limitations. Firstly, this preclinical pilot animal was a safety and operability evaluation of domestic EBV and thus sample sizes were small. Secondly, there was a need to increase the number of placed EBV due to the presence of collateral ventilation between the targeted lobe and the adjacent untargeted lobe, which may affect the results of this study. Lastly, although there are many similarities between pig and human lungs, animals in this study were not affected by emphysema, which would limit the applicability of domestic EBV. However, such a study was performed to provide a proof of concept that supports the use of domestic EBV products in clinical trials to treat patients with COPD.

## 5 Conclusion

Our results suggest that the EBV developed by Beijing Saishute Medical Devices Co., Ltd. is safe and reliable for placement in the bronchi of pigs. Its safety is similar to that of EBV produced by Pulmonx Corporation International, LLC, with good ease of use and operability. This product meets the safety evaluation requirements for animal testing. Our study is clinically significant as it provides an experimental basis for large-scale clinical research on domestic EBV products.

## Data availability statement

The original contributions presented in the study are included in the article/supplementary material, further inquiries can be directed to the corresponding authors.

## Ethics statement

The animal study was approved by Institutional Review Board of Shanghai Mincal Medical Research Co., Ltd. Large Animal Research Center (protocol code: ACU2021-BJSST-01). The study was conducted in accordance with the local legislation and institutional requirements.

## Author contributions

YJ: Writing – review & editing, Writing – original draft, Validation, Methodology, Investigation, Formal analysis, Data curation, Conceptualization. ST: Writing – review & editing, Writing – original draft, Investigation, Formal analysis, Data curation. JL: Writing – original draft, Validation, Methodology, Investigation. XS: Writing – original draft, Formal analysis. QW: Writing – review & editing, Data curation. XL: Writing – original draft, Visualization. WZ: Writing – original draft, Supervision, Project administration. YD: Writing – original draft, Supervision, Project administration. YL: Writing – review & editing, Project administration. CB: Writing – review & editing, Writing – original draft, Supervision, Project administration, Conceptualization. HH: Writing – review & editing, Writing – original draft, Validation, Methodology, Investigation, Formal analysis.
